# Sensitisation for cisplatin-induced apoptosis by isothiocyanate E-4IB leads to signalling pathways alterations

**DOI:** 10.1038/sj.bjc.6603434

**Published:** 2006-10-24

**Authors:** J Bodo, L Hunakova, P Kvasnicka, J Jakubikova, J Duraj, J Kasparkova, J Sedlak

**Affiliations:** 1 Laboratory of Tumour Immunology, Cancer Research Institute, Slovak Academy of Sciences, Vlarska 7, Bratislava 833 91, Slovakia; 2 Department of Nuclear Physics and Biophysics, Faculty of Mathematics, Physics and Informatics, Comenius University, Mlynska dolina F1, Bratislava 842 15, Slovakia; 3 Institute of Biophysics, Academy of Sciences of the Czech Republic, Kralovopolska 135, Brno CZ-61265, Czech Republic

**Keywords:** cisplatin, E-4IB isothiocyanate, apoptosis, cell cycle

## Abstract

A new synthetic isothiocyanate (ITC) derivative, ethyl 4-isothiocyanatobutanoate (E-4IB), appeared to be an effective modulator of cellular proliferation and potent inducer of apoptosis. In cooperation with cisplatin, this compound exerted synergistic effects in human ovarian carcinoma A2780 cells. In the present study we investigated in more detail E4IB-sensitisation for cisplatin-induced apoptosis. Sequential administration of both cytostatic agents led to increased intracellular platinum accumulation, glutathione level depletion and mitochondrial membrane potential dissipation. These events were accompanied with poly (ADP-ribosyl) polymerase cleavage, stimulation of caspase-3 activity, upregulation of p53, FasL and Gadd45*α*, cyclin B1 downregulation and an increase in mitogen-activated protein kinases JNK, ERK and p38 phosphorylation as well as PI3K level alterations. The presented results might have implications for developing new strategies aimed at therapeutic benefit of natural or synthetic ITCs in cooperation with various anticancer drugs.

Chemotherapy constitutes an essential approach of ovarian cancer treatment. Combined sensitiser/inducer concept in specific sequence, aimed at bringing tumour cell populations into a state where they are most susceptible to cytotoxic effects of chemotherapeutic agents, may be a novel strategy to enhance the efficacy of anticancer therapy in a variety of human cancers (Sampath and Plunkett, 2001; Schuchmann *et al*, 2006).

Cisplatin, one of the most potent antitumour agents with clinical activity against a variety of solid tumours, had changed the course of therapeutic management of these tumours (Zamble *et al*, 1998; Cohen and Lippard, 2001). Cytotoxicity of cisplatin is mediated by formation of DNA adducts as the key structures activating several signal transduction pathways including MAPKs resulting in activation of apoptosis (Siddik, 2003; Wang and Lippard, 2005). Intensive search for new compounds that could interfere with these pathways can help to increase platinum efficacy (Abou El Hassan *et al*, 2004; Wernyj and Morin, 2004; Rothermundt *et al*, 2006).

Naturally occurring isothiocyanates (ITCs) have been known to be effective chemopreventive agents (Steinmetz and Potter, 1991) and to exhibit a protective effect against cancers in a variety of target organs (Morse *et al*, 1993; Hecht, 1995). Isothiocyanates perturb several steps in the carcinogenic process by inhibiting cell growth due to cell cycle arrest and removing premalignant and malignant cells through the activation of apoptosis (Gamet-Payrastre *et al*, 2000; Miyoshi *et al*, 2004; Pullar *et al*, 2004).

In the previous study (Bodo *et al*, 2006), we introduced a new synthetic ITC-derivative, ethyl 4-isothiocyanatobutanoate (E-4IB), as an efficient modulator of cell cycle and apoptosis. In search for novel strategies to sensitise tumour cells, the objective of this study was to describe further activities of E-4IB to render cisplatin-induced apoptosis in human ovarian tumour cell line A2780. We indicated that this ITC-derivative enhanced intracellular platinum accumulation and conferred the cells a state of increased responsiveness to cisplatin-induced apoptosis associated with alterations in cell cycle protein regulation, glutathione (GSH) levels, caspase-3 activation, mitochondrial potential and signalling pathways including MAPKs family members.

## MATERIALS AND METHODS

### Reagents

E-4IB was synthesised as described (Floch *et al*, 1997). Dimethyl sulphoxide (DMSO), RNase A, monochlorobimane (MCB) and propidium iodide (PI) were obtained from Sigma Chemical Co. (St Louis, MO, USA). Cisplatin was acquired from LACHEMA (Brno, Czech Republic). Rabbit polyclonal antibodies against ERK1/2, phospho-ERK1/2, JNK1/2, p38, FasL, actin, PARP, p21, p53, Gadd45*α*, cyclin B1 and horseradish peroxidase-conjugated anti-rabbit antibody were obtained from Santa Cruz Biotechnology (Santa Cruz, CA, USA). Rabbit polyclonal anti-ACTIVE JNK- and anti-ACTIVE p38 antibodies were purchased from Promega (Medison, WI, USA). Cationic JC-1 dye was a product of Molecular Probes (Eugene, OR, USA). Caspase substrate (Ac-DEVD) and caspase inhibitor (Z-DEVD) were gained from Alexis (Lausen, Switzerland).

### Cell culture, drug treatments, clonogenic assay and apoptosis

Human ovarian carcinoma cells A2780 were routinely cultured in RPMI 1640 medium supplemented with 10% heat-inactivated FCS, 2 mM L-glutamine, 100 *μ*g ml^−1^ penicillin and 50 *μ*g ml^−1^ streptomycin. 0.5 × 10^6^ cells ml^−1^ were cultured in 96-, 24- or six-well plates (Greiner, Germany). Cells were incubated with E-4IB or cisplatin at indicated concentrations and time determined by MTT assay (Bodo *et al*, 2005). In combination, we used sequential treatments with E-4IB followed by cisplatin, when E4IB was added 3 h before cisplatin. Clonogenic growth after treatments for 7 days was assessed by Giemsa-Romanowski staining and measured using ImageJ 1.33u software (National Institutes of Health, USA). Apoptosis was assessed by flow cytometric analysis of PI-stained nuclei as previously described (Jakubikova *et al*, 2005).

### Measurements of platinum accumulation

Flameless atomic absorption spectrophotometry (FAAS) was used to analyse platinum levels in tested cells. Cultures were digested in nitric acid followed by H_2_O_2_ and HCl addition. Flameless atomic absorption spectrophotometry measurements were performed with a Varian AA240Z Zeeman atomic absorption spectrometer equipped with a GTA 120 graphite tube atomizer (Crul *et al*, 2001; Pluim *et al*, 2004).

### GSH assay

Cells were seeded in 96-well microplates (black wells with transparent bottom) at a density of 5 × 10^3^ well^−1^ and allowed to adhere in the growth medium to 50–60% confluence before being exposed to the tested drugs and buthionine-sulphoximine (BSO), respectively, for 3, 6 or 27 h in quadruplicates. After incubation with MCB (40 *μ*M) in the dark, at room temperature for 20 min and several washings, samples were measured at 390/520 nm (excitation/emission) using the POLARstar fluorimeter (BMG Labtech GmbH, Germany) (Kamencic *et al*, 2000).

### Caspase-3 activity and mitochondrial potential determination

Measurements of caspase-3 activity were performed with the use of fluorescence technique as described (Moriya *et al*, 2000). Briefly, 3 × 10^6^ washed cells were lysed in a buffer (pH 7.5) containing 50 mM TRIS, 1 mM EDTA, 10 mM EGTA, 0.5 % Triton X-100 and 1 mM DTT for 10 min and samples were centrifuged at 10.000 **g** for 10 min. Cleared lysates (10 *μ*g of protein) containing 50 *μ*M of substrate were incubated at 37°C for 1 h. An amount of released AFC (7-amino-4-trifluoromethyl coumarin) was measured with the POLARstar fluorimeter using excitation at 390 nm and emission at 520 nm.

Mitochondrial membrane potential (*Ψ*_m_) was assessed by JC-1 dye measurement of red/green fluorescence ratio as described (Jakubikova *et al*, 2005). Briefly, cells were washed twice with PBS and incubated with 400 *μ*l of PBS/0.2% BSA containing 4 *μ*M of JC-1, at 37°C for 30 min. Fluorescence was measured by Coulter Epics Altra flow cytometer. Data were analysed by the WinMDI version 2.8 software (J Trotter, Scripps Research Institute, La Jolla, CA, USA).

### Western blot analysis

To examine effects of E-4IB and cisplatin on membrane protein expression, A2780 cells were harvested at indicated times, lysed in a buffer as described (Bodo *et al*, 2006). For each lane 50 *μ*g of protein were loaded. Blots were incubated with the indicated antibodies and horseradish peroxidase-conjugated anti-rabbit secondary antibody (1 : 3000 diluted). All primary antibodies were used at final concentration 1 *μ*g ml^−1^. Enhanced chemiluminescence (ECL, Amersham Arlington Heights, IL, USA) was used for detection. Expression of actin was used as a control for equal gel loading.

### Statistical analysis

Statistical significance was analysed by Analysis of variance (ANOVA) and *post hoc* (Tukey) tests.

## RESULTS

### Cell sensitisation and growth inhibition

In the recent paper (Bodo *et al*, 2005), we referred relatively low cytotoxic activity of synthetic ITC E-4IB in human ovarian A2780 cells and tested it in combination with conventional cytotoxic drug cisplatin. To study experimental conditions, we fixed treatment dose- and time-schedule (2.5, 5 *μ*M E-4IB; 5, 10 *μ*M cisplatin, for 6 or 27 h) and found that pretreatment of cisplatin with E-4IB (E-4IB/cisplatin) induced apoptosis in a dose- and time-dependent manner. We also observed that sequential treatment with E-4IB followed by cisplatin yielded more apoptotic cells than cotreatment with both compounds. In the present study we showed that E-4IB cooperated with cisplatin to inhibit clonogenic tumour cell growth (Table 1). To study E-4IB and cisplatin effects, experiments revealed that cellular accumulation of platinum in combination with E-4IB (E-4IB/cisplatin) is more than three-fold higher (9.28±0.69 ng Pt/10^6^ cells) than that observed with a single cisplatin (2.97±0.32 ng Pt/10^6^ cells) (Table 2).

### GSH level alterations

**Figure 1:**
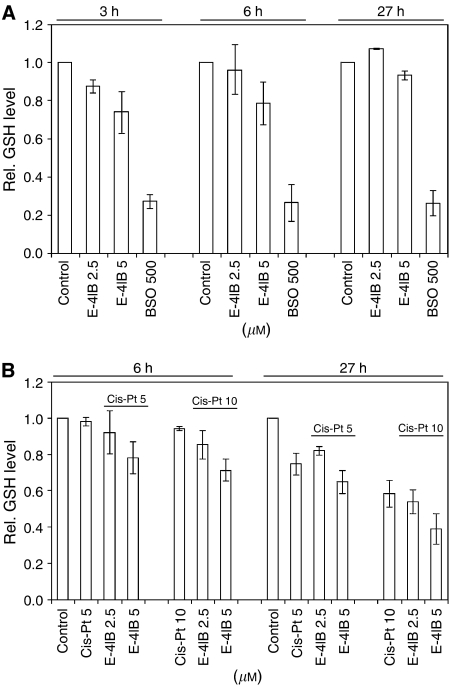
(**A**) Modulation of GSH levels after E-4IB treatment. A2780 cells were exposed to 2.5 and 5 *μ*M E-4IB for 3, 6 and 27 h. BSO (500 *μ*M) was used as a control for GSH inhibition. (**B**) Cisplatin or E-4IB/cisplatin-induced alterations in GSH levels. A2780 cells were exposed to E-4IB (2.5 or 5 *μ*M) for 3 h followed by cisplatin (5 or 10 *μ*M) for 3 or 24 h. The results shown are representative of at least three independent experiments.

In ovarian cancer cells, increased GSH levels have been correlated directly with the drug resistance (Okuno *et al*, 2003). We investigated therefore the capacity of A2780 ovarian cancer cells, treated cells with E-4IB and/or cisplatin, to regulate GSH. Experiments aimed at the testing of a single drug or sequential treatments with E-4IB followed by cisplatin showed GSH decrease in a dose- and time-dependent manner. Glutathione inhibition by BSO was utilised as control (Figure 1A and B).

### Caspase-3 activity and mitochondrial potential *Ψ*_m_

**Figure 2:**
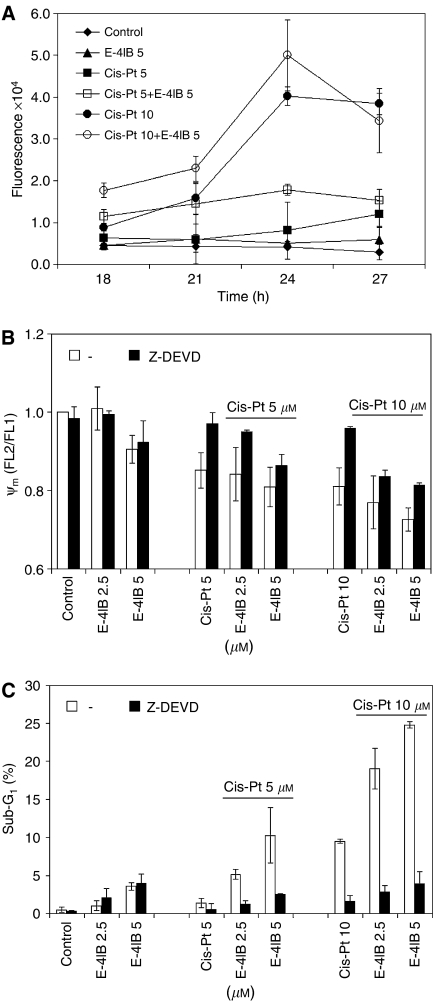
(**A**) Activation of caspase-3 after E-4IB and/or cisplatin treatment in A2780 cells. The cells were exposed to E-4IB (5 *μ*M) for 3 h followed by cisplatin (5 or 10 *μ*M). Caspase-3 activity was measured using fluorogenic substrate Ac-DEVD-AFC. (**B**) Modulation of *Ψ*_m_. Caspase-3 inhibitor (Z-DEVD) was added to culture medium for 1 h before the exposure of the cells to 2.5 or 5 *μ*M E-4IB for 3 h followed by cisplatin (5 or 10 *μ*M, for 3 or 24 h). (**C**) Effect of Z-DEVD on the sub-G_1_ population in the cells treated by E-4IB and/or cisplatin. Sub-G1 was calculated by flow cytometry. The results are representative of at least three independent experiments.

Fluorescence substrate (Ac-DEVD-AFC) detection test was used to see whether sensitisation by E-4IB followed by cisplatin involves caspase-3 activation. We found that caspase-3 is involved in a single cisplatin treatment and its activity is increased at E-4IB/cisplatin combination in a dose- and time-dependent manner (Figure 2A). In addition, caspase-3 inhibition Z-DEVD technique was used for the quantification of Δ*Ψ*_m_. Incubation of cells in the presence of E-4IB/cisplatin caused a significant reduction in *Ψ*_m_ in a dose-dependent manner and this was inhibited by Z-DEVD (Figure 2B). Finally, we analysed the involvement of caspase-3 in E-4IB sensitisation using Z-DEVD inhibitor. As observed by detection of sub-G_1_ population measured by flow cytometry, Z-DEVD significantly reduced E-4IB/cisplatin-induced apoptosis (Figure 2C).

### Modulation of apoptosis regulatory molecules

**Figure 3:**
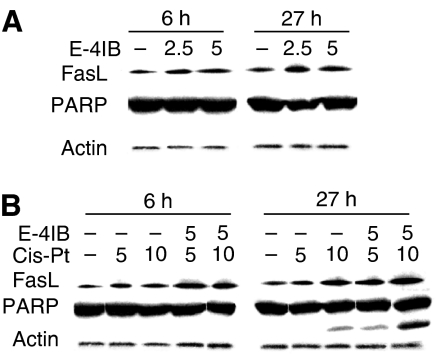
Western blots of apoptosis-related proteins FasL and PARP. A2780 cells were exposed to E-4IB (**A**) or cisplatin or E-4IB/cisplatin (**B**). Experimental conditions were used as shown in Figure 1. Western blot analysis was performed with total cell extracts. Fifty micrograms of cell lysate was applied for FasL or PARP expression. Actin was utilised for equal protein loading.

To gain further inside into the molecular mechanism(s) of apoptosis regulation upon treatment with the studied drugs by Western blot analysis, we inspected several key molecules associated with these events. Treatment with a single E-4IB resulted in upregulation of FasL, but no PARP cleavage appeared (Figure 3A). However, E-4IB significantly enhanced the ability of cisplatin to induce PARP cleavage. Additionally, E-4IB/cisplatin treatments led to the enhancement of FasL levels which culminated at 5 *μ*M E-4IB/10 *μ*M cisplatin concentrations (Figure 3B).

### Modulation of cell cycle regulation proteins

**Figure 4:**
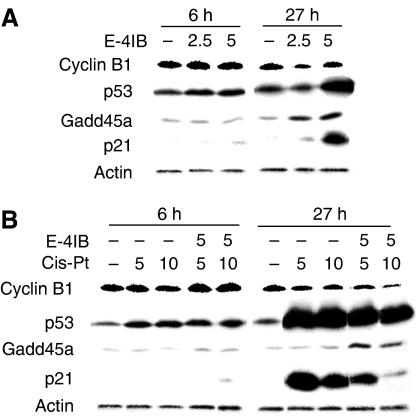
Western blot analysis of A2780 cell protein extracts after E-4IB (**A**), or cisplatin or E-4IB/cisplatin treatments (**B**). Cyclin B1, p53, p21 and Gadd45*α* protein levels were evaluated. Experimental conditions were used as shown in Figure 1.

To study cell cycle protein alterations, we examined the levels of cell cycle-responsive molecules such as cyclin B1, p53, p21 and Gadd45*α* (growth arrest- and DNA damage-inducible protein). Ovarian cells were exposed to E-4IB (Figure 4A) and/or cisplatin (Figure 4B) and visualised by Western blotting. P53, p21 and Gadd45*α* levels were enhanced by a single E-4IB treatment in a time- and dose-dependent manner. On the contrary, cyclin B1 levels significantly dropped after E-4IB or E-4IB/cisplatin treatments. Interestingly, after prolonged time (27 h), abundant amounts of p53 after cisplatin or E-4IB/ciplatin treatments were determined. However, p21 levels culminated at the concentration of 5 *μ*M cisplatin and then continuously decreased due to increased cisplatin or E-4IB/cisplatin concentrations. Surprisingly, single cisplatin treatments exerted no alterations in Gadd45*α* levels, but these remained at similar levels as observed with a single E-4IB (Figure 4A and B).

### Protein kinases alterations

**Figure 5:**
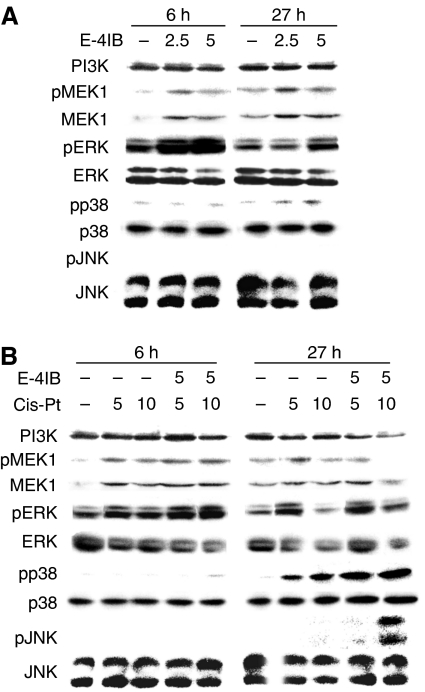
Western blot analyses of E-4IB (**A**) or cisplatin or E-4IB/cisplatin (**B**). ERK1/2, pERK1/2, JNK1/2, pJNK1/2, p38, pp38, MEK1, pMEK1 and PI3K protein levels were determined. Cells were treated as shown in Figure 1.

Alterations in the activities of the MAPKs and PI3K levels are generally supposed to be associated with the control of cell proliferation, differentiation and death (Wada and Penninger, 2004). In this context, after extracellular stimuli with E-4IB and/or cisplatin, we assessed MAPKs activities to be linked with phosphorylation and also measured PI3K alterations. Single E-4IB administration (Figure 5A) exerted no influence on constitutive ERK1/2 levels. However, upregulation of both constitutive and phosphorylated MEK1, in a dose-dependent manner, was observed and this correlated with an enhancement of ERK1/2 phosphorylation. Interestingly, no substantial alterations in constitutive JNK1/2 and p38 MAPK were determined, whereas phosphorylated p38 MAPK form rose. Contrariwise, 27 h E-4IB/cisplatin treatment was linked to an enhanced phosphorylation of both JNK and p38 MAPK. Conversely, MEK1, pMEK1, ERK1/2 and PI3K (phosphatidylinositol 3-kinase) contents were lowered (Figure 5B).

## DISCUSSION

The major mode of cisplatin effect is mediated by interaction with DNA to form DNA adducts and activation several signal transduction pathways that culminate in a variety of tumour cell lines in the cell growth arrest and apoptosis. In chemotherapy cisplatin was utilised to cooperate with several anticancer compounds to enhance efficacy of its antitumour activity generally resulting in apoptosis (Reed, 1998; Roberts *et al*, 2005; Siegel-Lakhai *et al*, 2005).

In search for strategy to study several natural and synthetic compounds in chemotherapy we (Bodo *et al*, 2005) and others (Fimognari *et al*, 2006) recently referred activities of ITCs as ‘sensitizers’ followed by conventional chemotherapeutic compounds as ‘inducers’, to trigger inhibition of proliferation and their synergy to stimulate apoptosis in cancer cells. We further showed that the cytostatic activity of E-4IB was more pronounced than its cytotoxic activity at equimolar concentrations. In the present study, using the same experimental system, we confirmed this synergy by clonogenic assay and investigated for the first time signalling pathways due to their cooperative effect. We determined that E-4B/cisplatin-induced apoptosis correlated with enhanced intracellular platinum accumulation. These events were associated with GSH depletion, downregulation of cyclin B1, mitochondrial potential dissipation, PARP cleavage, p53-, Gadd45*α*- and p21-upregulation, attesting to the specific effect of E-4IB sensitisation for cisplatin-induced cell cycle alterations and apoptosis.

The mechanisms of cellular uptake and efflux of cisplatin are still not fully understood. However, it was documented that intracellular GSH and exporter proteins have an important role in those processes, presumably by drug inactivation and drug efflux promotion (Mistry *et al*, 1993; Wang and Lippard, 2005). In the present study we found reduction of intracellular GSH in a dose-dependent manner after E-4IB treatments. Thus, observed increase of platinum intracellular accumulation stimulated by E-4IB pretreatments could be explained by a decrease of GSH availability to form cisplatin conjugates and thereby subsequent reduction of cellular efflux of the drug.

It has been shown that activation of JNK leads to targeting of the activator protein-1 (AP-1) transcription factor complex binding sites in the promoters of multiple target genes such as FasL, an element of death receptor-dependent apoptotic signalling cascade (Le Niculescu *et al*, 1999). In this context, combination E-4IB and cisplatin in our experimental conditions led to marked enhancement in FasL levels. Thus, we also assume that FasL may play an important role in initiation of E-4IB/cisplatin-induced apoptosis.

The molecular mechanisms for cisplatin-induced apoptosis involved caspase-3 activation (Kolfschoten *et al*, 2002). To see an importance of caspase-3, we inspected the activity of the enzyme and demonstrated a time- and dose-dependent activation of caspase-3 caused by single drug treatments or in combination. Flow cytometric analysis aimed at the regulation of mitochondrial potential and an appearance of sub-G1 portion of apoptotic cells confirmed an essential role of this enzyme in E-4IB/cisplatin-induced apoptosis.

Our results further showed marked increase in p53 levels after cisplatin treatment, which, surprisingly, were not affected by E-4IB sensitisation. These events were after E-4IB/cisplatin treatments associated with moderate increase in Gadd45*α* protein levels. This may suggest that E-4IB, concomitantly with Gadd45*α* (growth arrest- and DNA damage-inducible protein), might contribute to the transcription activation of p53. In fact, in response to genotoxic stimuli, Gadd45*α* was shown to be increased and participated in nucleotide excision repair thereby it sensitised different tumours to undergo apoptosis (Sheikh *et al*, 2000). P53 and cdk inhibitor p21 have also been determined to maintain G_2_ arrest following DNA damage (Taylor and Stark, 2001). In addition, some experimental data show that p21 blocks cell cycle progression through its negative activity on various cyclin-dependent kinases and apoptosis through inhibition of caspases activation (Viallard *et al*, 2001). These events involve an initial inhibition of cyclin B1/Cdc2 activity by p21 and a subsequent reduction of cyclin B1 (Charrier-Savournin *et al*, 2004). In relation to the reduction of cyclin B1 observed in our experiments we assume that the diminution of p21 observed after E-4IB/cisplatin combination treatments facilitate the cells to trigger apoptosis.

Three MAPK members (JNK, ERK and p38) participate in integrating extracellular signals to regulate cell proliferation, differentiation, cell survival and apoptosis (Dent and Grant, 2001). The kinases were activated following exposure of tumour cells to cisplatin, when ERK activation might be critical for cisplatin-induced apoptosis (Persons *et al*, 2000; Yeh *et al*, 2002). Furthermore, the duration of JNK- and p38 MAPK activation was demonstrated as an early key determinant of cisplatin-induced apoptosis (Sanchez-Perez *et al*, 1998; Benhar *et al*, 2001; Mansouri *et al*, 2003; Kusama *et al*, 2006). In accordance with these results we presented here sustained activation/phosphorylation of p38 MAPK and this was associated with E-4IB/cisplatin-induced apoptosis.

Phosphatidylinositol 3-kinase is believed to be important cell survival factor in human ovarian cancer cells (Asselin *et al*, 2001). PI3K/AKT pathway facilitates G_1_ to S phase progression and plays a major role in protecting of cells from apoptosis by inhibiting Bad and thus cytochrome *c* release, inactivating caspase-3 and targeting p53 for its destruction (Kennedy *et al*, 1997). In our experimental system we found continuous downregulation of PI3-kinase and, in concordance with others, we assume that these events facilitate cell cycle arrest and apoptosis as a consequence of E-4IB/cisplatin treatment.

The presented results indicate the potential of E-4IB for anticancer therapy may largely reside in its ability to sensitise tumour cells to overcome cisplatin resistance and to induce apoptosis. Thereby, our studies showed potential application of E-4IB as sensitiser for cytotoxic therapies in ovarian cancer cells. Finally, in terms of clinical perspective, the combined sensitiser (E-4IB)/inducer (cisplatin) strategy may be a novel approach to enhance the efficacy of anticancer therapy in some human cancers.

## Figures and Tables

**Table 1:** Effect of E-4IB on cisplatin-induced clonogenic survival

**Concentration (*μ*M)**		**Relative average**
**E-4IB**	**Cisplatin**	**Count**
—	—	1.00±0.00
0.5	—	0.91±0.06
1	—	0.79±0.09
		
—	0.1	0.74±0.05^*^
0.5	0.1	0.64±0.11
1	0.1	0.40±0.02^#^
		
—	0.25	0.43±0.08^**^
0.5	0.25	0.32±0.02
1	0.25	0.10±0.09^#^

E-4IB=ethyl 4-isothiocyanatobutanoate.

A2780 cells were treated with 0.1 or 0.25 *μ*M cisplatin in the presence or absence of 0.5 or 1 *μ*M E-4IB. Clonogenic survival was assessed by Giemsa-Romanowski staining after 7 days and colony numbers per well of a six-well plate are indicated. Statistical significant differences from the controls, ^*^P<0.05, ^**^P<0.01, and from the corresponding cisplatin-treated samples, ^#^*P*<0.05.

**Table 2:** Effect of E-4IB on platinum intracellular accumulation

**Samples**	**Control**	**E-4IB**	**Cisplatin**	**E-4IB+cisplatin**
ng Pt/10^6^ cells	0.29±0.04	0.34±0.13	2.97±0.32^**^	9.28±0.69^##^

E-4IB=ethyl 4-isothiocyanatobutanoate; FAAS=flameless atomic absorption spectrophotometry.

A2780 cells were treated with 10 *μ*M cisplatin in the presence or absence of 5 *μ*M E-4IB. Flameless atomic absorption spectrophotometry was used to analyse platinum levels. Statistical significant differences from the controls, ^**^P<0.01, and from the cisplatin-treated samples, ^##^*P*<0.01.
